# ARTFLOW: A Fast, Biologically Inspired Neural Network that Learns Optic Flow Templates for Self-Motion Estimation

**DOI:** 10.3390/s21248217

**Published:** 2021-12-08

**Authors:** Oliver W. Layton

**Affiliations:** Department of Computer Science, Colby College, Waterville, ME 04901, USA; oliver.layton@colby.edu

**Keywords:** optic flow, heading, self-motion, neural decoding, adaptive resonance theory, vision, biologically inspired, neural network

## Abstract

Most algorithms for steering, obstacle avoidance, and moving object detection rely on accurate self-motion estimation, a problem animals solve in real time as they navigate through diverse environments. One biological solution leverages optic flow, the changing pattern of motion experienced on the eye during self-motion. Here I present ARTFLOW, a biologically inspired neural network that learns patterns in optic flow to encode the observer’s self-motion. The network combines the fuzzy ART unsupervised learning algorithm with a hierarchical architecture based on the primate visual system. This design affords fast, local feature learning across parallel modules in each network layer. Simulations show that the network is capable of learning stable patterns from optic flow simulating self-motion through environments of varying complexity with only one epoch of training. ARTFLOW trains substantially faster and yields self-motion estimates that are far more accurate than a comparable network that relies on Hebbian learning. I show how ARTFLOW serves as a generative model to predict the optic flow that corresponds to neural activations distributed across the network.

## 1. Introduction

When it comes to navigation through diverse, complex environments, animals demonstrate capabilities that far outpace those of machines. Principles derived from biological systems have the potential to catalyze efficient solutions that enhance autonomy in unmanned aerial vehicles (UAVs) and other mobile robotic systems [1,2,3,4]. Notably, many animals rely primarily on vision to perceive their self-motion in just fractions of a second. Insects [5,6] and humans [7] use a rich source of visual information about self-motion to guide navigation known as optic flow, the pattern of motion that arises on the eye. Optic flow holds promise for UAV navigation because computing it only requires a single-resolution camera sensor of modest spatial resolution [8]. This could enable light-weight, energy-efficient solutions that reduce or eliminate the need for LiDAR, binocular cameras, and other large sensor arrays. Since optic flow based navigation does not require external communication, it could increase autonomy in situations where GPS signals or radio connections are unreliable or absent.

**Figure 1:**
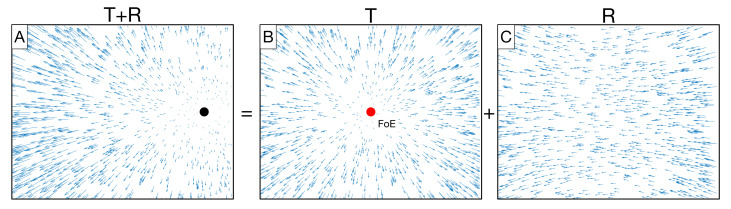
The optic flow experienced by a moving observer (**A**) is the sum of translational (**B**) and rotational (**C**) components. The motion singularity in the presence of rotation (black disk) need not correspond to the FoE (red disk), which corresponds to the heading direction of the observer. Heading of 0 corresponds to straight-ahead (**B**), and headings to the right (left) are positive (negative). The influence of yaw rotation is depicted in (**A**).

Mathematically, any first-order optic flow pattern that corresponds to self-motion through a rigid environment can be decomposed into components that represent the translation and rotation of the observer (Figure 1) [9]. The translational component consists of motion that radiates from a singularity known as the focus of expansion (FoE) (Figure 1b) [10]. The rotational component (Figure 1C) reflects changes in the 3D orientation of the observer (pitch, yaw, and roll) when movement occurs along a straight path. When self-motion does not involve rotation, the position of the FoE specifies the observer’s instantaneous direction of movement (heading). As Figure 1 illustrates, however, the presence of rotation may cause the position of the singularity to shift such that it no longer corresponds to the heading direction (compare Figure 1A and Figure 1B). Solutions to this problem tend to exploit visual [11,12,13,14] or nonvisual [15,16,17] information to estimate the rotational component (*R*) and factor it out from the raw optic flow pattern (T+R). This yields the translational component (T=T+R−R) from which heading may be estimated.

While numerous computer vision algorithms estimate self-motion from optic flow [18], the focus here is on biologically inspired approaches that emulate mechanisms in the brain. A large family of approaches models the structure and function of the medial temporal (MT) and dorsal superior temporal (MSTd) areas of the primate brain [11,19,20,21,22,23,24]. Neurons in MT demonstrate sensitivity to the direction and speed of motion within small regions of the visual field. MSTd contains neurons that demonstrate tuning to full-field radial and laminar patterns of motion that resemble translational (Figure 1B) and rotational (Figure 1C) optic flow, respectively [25,26]. Consistent with neurophysiology, each model MSTd neuron performs a feedforward integration of local motion signals from MT in a configuration that matches the preferred optic flow pattern of the MSTd cell. For example, a cell sensitive to the pattern of radially expanding optic flow that corresponds to self-motion along a straight-ahead heading (0; Figure 1B) would receive leftward motion signals on the left side of the visual field, rightward motion signals on the right side of the visual field, etc. This process can be thought of as matching optic flow signals with motion pattern “templates.” The preferred heading of each MST cell is determined by the position of the FoE in its template (e.g., 0 for a template that resembles Figure 1B). The overall heading estimate considers the activation of all model MSTd cells. For example, the heading preference of the maximally active cell or a weighted sum of the preferences of active cells could constitute the estimate.

Many models rely on predefined templates that necessarily make assumptions about the layout of the environment to match optic flow signals [11,19,21,22,23,24,27,28,29]. For example, radial templates (Figure 1B) correspond to linear self-motion toward the fronto-parallel wall, ground templates assume that the observer moves a fixed height over a flat ground plane, etc. Actual self-motion scenarios inevitably deviate from these layouts. Even if the predefined templates yield reasonable heading estimates in some cases [11,30], it is difficult to anticipate how well they will generalize and support accurate self-motion estimates. Moreover, the set of predefined templates may not efficiently capture the range of optic flow patterns encountered during navigation through diverse, novel environments.

### Learning Optic Flow Templates for Self-Motion Estimation

Here I present a fast, biologically inspired neural network that learns optic flow templates that can be used to estimate self-motion through arbitrary environments. The approach builds on the fuzzy adaptive resonance theory (fuzzy ART) family of biologically inspired unsupervised learning algorithms [31,32]. Fuzzy ART leverages a combination of feedforward, recurrent, and feedback neural signals during learning to update existing learned representations only when a close enough match is obtained to the current sample, which results in stable learning. The proposed network, ART Fuzzy Logic Optic flow Wayfinder (ARTFLOW), inherits the following compelling advantages of fuzzy ART networks over those that rely on the backpropagation algorithm [33]:

- Learned representations are stable and do not suffer from catastrophic forgetting.
- The learning process and predictions are explainable.
- Effective learning is possible with only a single pass through training samples (one-shot learning).
- Lifelong learning: the learning process need not occur in discrete training and prediction phases—learning may continue during operation.

Building on these strengths, I made contributions in two key areas to make the fuzzy ART neural network suitable for learning optic flow and other sensory patterns.

**Figure 2:**
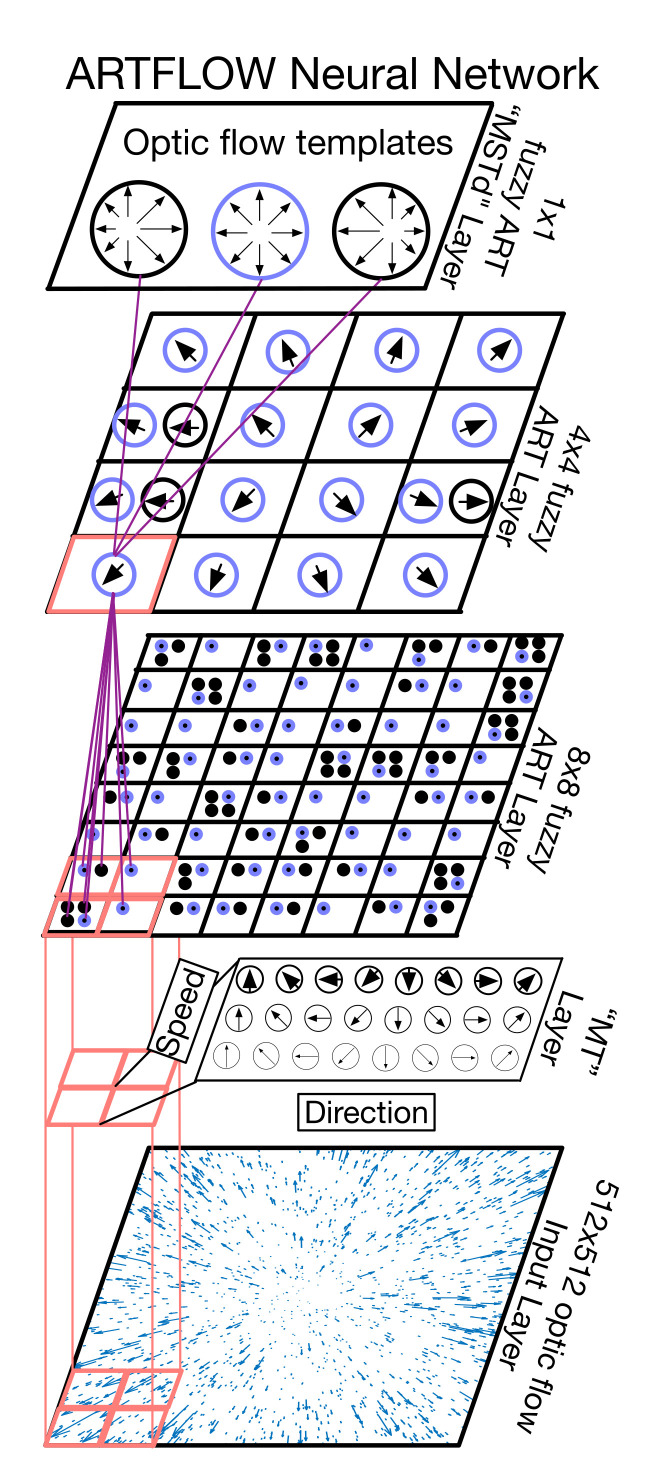
Overview of the ARTFLOW neural network architecture. The hierarchical network learns optic flow template patterns (top layer) from sequences of optic flow. Depicted is an example 3-layer network configured with 8 × 8, 4 × 4, and 1 × 1 grids of fuzzy ART modules, respectively. The MT layer preprocesses the optic flow, embedding each local signal into a neural representation with respect to motion speed and direction. Black squares represent distinct fuzzy ART modules, and circles in each fuzzy ART layer represent “committed cells” in the fuzzy ART network contained within each module. Black (blue) circles illustrate inactive (active) neurons. Committed cells learn distinct patterns based on the optic flow statistics that appear within each receptive field. Hence, the number may naturally vary across modules, as depicted by the different number of circles in the squares. Only connections corresponding to processing of the optic flow in the bottom-left corner are shown (pink squares). Each module is fully connected (purple lines) to committed cells in one or more nearby modules in the previous layer.

First, neurons are fully connected in typical fuzzy ART neural networks. Analogous to multi-layer perceptrons (MLPs), such dense connections do not scale well to the large numbers of features encountered when processing sensory data. Additionally, spatial relationships among features are not preserved. This is problematic for processing optic flow wherein the configuration of the motion vectors in the global pattern is important. I addressed this challenge in ARTFLOW by introducing parallel fuzzy ART modules that tile space on a discrete grid (Figure 2). Each module only processes signals that arise within its limited receptive field (RF). This local processing architecture bears similarities to convolutional neural networks (CNNs), which excel at processing sensory data.

Second, fuzzy ART consists of strictly two network layers, which does not afford learning of hierarchical relationships among features at multiple scales. In ARTFLOW, the outputs of topographically close, spatially offset fuzzy ART modules converge in the next network layer (Figure 2). Similar to CNNs, this gives rise to emergent features at different hierarchical levels. Unlike CNNs, however, learned features are spatially confined to the portion of the visual field sampled by each fuzzy ART module. That is, weights are not shared between modules. For example, modules sampling the bottom of the visual field learn optic flow patterns that reflect the statistics of ground flow while those that sample the top of the visual field learn distinct representations reflecting flow from trees, ceilings, etc.

I evaluated the ARTFLOW network using video datasets of simulated self-motion through dot-defined environments and visually realistic neighborhood and warehouse scenes rendered using the Unreal video game engine. The dot-defined scenes serve as controlled environments with which to test learning on large numbers of optic flow patterns that arise during specific types of self-motion. I decoded activations from the top “MSTd” layer of ARTFLOW to assess how well the learned optic flow templates support accurate heading estimation. I compared performance to an identically structured hierarchical network that replaces fuzzy ART modules with those that rely on the simpler Hebbian learning law.

## 2. Materials and Methods

### 2.1. Optic Flow Datasets

#### 2.1.1. Dot-Defined Environments

**Figure 3:**
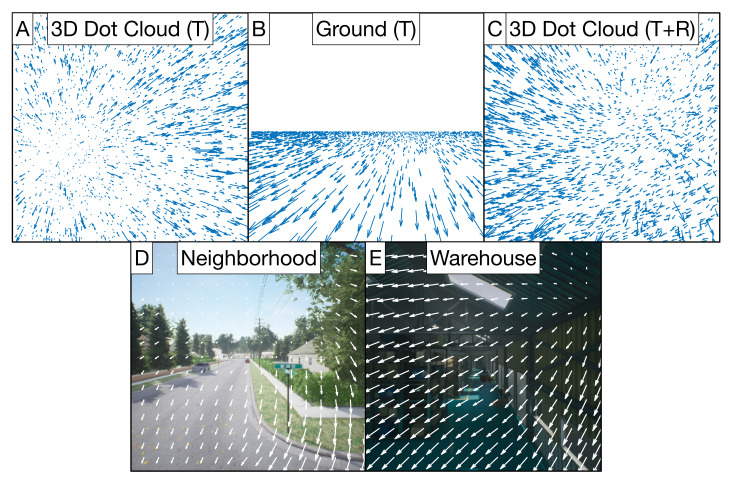
Example optic flow samples from the random dot (**top** row) and Unreal (**bottom** row) datasets. Samples consisted of translational optic flow, except for those in the 3D Dot Cloud (T+R) dataset, which also contained rotation. One such sample is shown in (**C**) with $\left(5\mathrm{azimuth},1\mathrm{elevation}\right)$ translation, and 7 /s yaw and roll rotation.

Table 1 summarizes the camera parameters used to generate the datasets of simulated self-motion through the two environments that consist of randomly positioned dots. In these environments, I distributed the dots either along a ground plane or within a 3D cloud in front of the observer. Each sample in the dataset consisted of self-motion along a constant linear path for 10 digital video frames, 0.33 s in duration with a 30 frames-per-second frame rate. On each video frame, I clipped and replaced dots that exited the field of view or valid depth range to ensure that the same number of dots always remained visible. I computed the optic flow using a pinhole camera model [18] and standard analytic equations [9]. This served as the input to the ARTFLOW network. Figure 3 shows example optic flow fields from 3D dot cloud and ground environments. Table 2 specifies the conditions used to generate the datasets in each simulation experiment.

#### 2.1.2. Neighborhood and Warehouse Environments

The neighborhood and warehouse datasets were generated using Microsoft AirSim [34], a simulation environment for drones that renders visually realistic scenes using the Unreal game engine. The neighborhood dataset consists of self-motion through a rich outdoor scene with grass, trees, houses, fences, streets, and other objects. The warehouse environment consisted of a darker indoor scene with tall shelving, skylight reflections, and boxes. I created 150 ten frame video samples from each of these environments at 512 × 512 resolution. I estimated the optic flow from the videos using DeepFlow2 [35]. Figure 3 shows example optic flow fields extracted from the neighborhood and warehouse scenes.

### 2.2. Overview of ARTFLOW Neural Network

**Figure 4:**
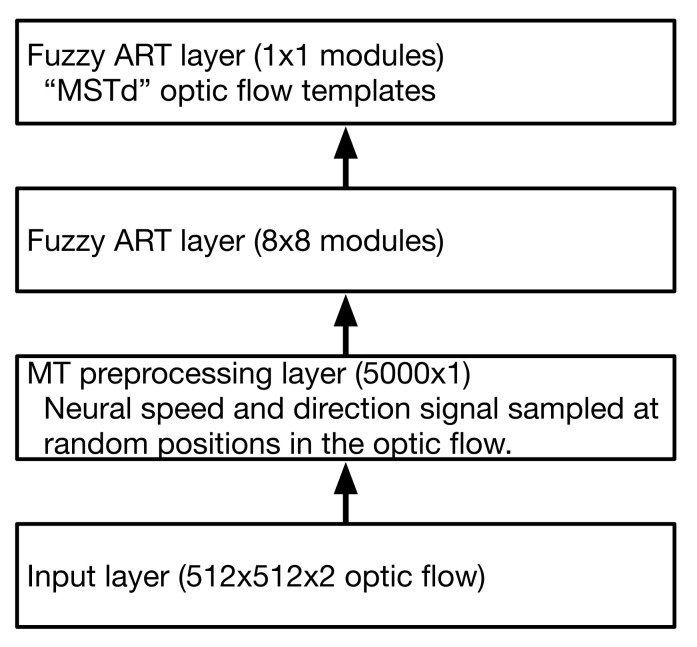
ARTFLOW network configuration used in simulations.

Figure 4 shows the configuration of the two-layer ARTFLOW neural network used in simulations. The first non-input layer embeds optic flow inputs into a neural representation that emulates primate area MT (MT preprocessing layer). This yields a distributed code of the optic flow with respect to speed and direction. Subsequent layers contain separate modules arranged in a spatial, non-overlapping grid (henceforth “fuzzy ART layer”). Each module implements an independent instance of the fuzzy ART neural network (henceforth “fuzzy ART module”). The number of fuzzy ART layers and connectivity between them is flexible. For example, Figure 2 shows a three layer network with 8 × 8, 4 × 4, and 1 × 1 module configurations in each respective layer. I found that two layers were sufficient for the simulations reported here. Connections between modules in successive layers followed a hierarchical, “fan in” configuration—the first fuzzy ART layer consisted of an 8 × 8 grid of modules that converged to a single module in the second fuzzy ART layer. The weights of neurons in this top layer represent the learned optic flow templates.

Table 3 summarizes hyperparameters and their default values in ARTFLOW.

### 2.3. MT Preprocessing Layer

Model MT neurons exhibit sensitivity to the speed and direction of optic flow signals over time. I based parameter values on known neurophysiology where possible.

#### 2.3.1. Optic Flow Integration

I positioned 5000 ($N_{MT}$) model MT neurons such that they sampled random portions of the 512 × 512 pixel optic flow field. Each neuron integrated motion within its 15 pixel radius (≈5 diameter) circular RF [36].

Direction preferences were uniformly distributed. Tuning to the direction θ(x,y) present at position (x,y) within the RF of a neuron relative to the preferred direction $\theta_{pref}$ followed a von Mises distribution:

(1) $$d_{MT}\left(x,y;\theta_{pref}\right)=exp\left(\sigma_{\theta }\left(cos\left(\theta \left(x,y\right)-\theta_{pref}\right)-1\right)\right)$$

where $\sigma_{\theta }=3$ indicates the bandwidth of the direction tuning, which was set to approximate the ≈90 full-width at half-maximum found in MT cells [37,38].

The tuning of each model MT neuron to the optic flow speed (ν) at position (x,y) within the RF followed a log-normal distribution [39]:

(2) $$s_{MT}\left(x,y;\nu_{pref}\right)=exp\left(-\frac{log{\left(\frac{\nu \left(x,y\right)+s_{0}}{\nu_{pref}+s_{0}}\right)}^{2}}{2\sigma_{\nu }^{2}}\right)$$

where $\sigma_{\nu }$ defines the speed tuning bandwidth; $s_{0}$ defines a non-negative offset parameter to prevent the singularity in the logarithm at 0; and $\nu_{pref}$ defines the preferred speed of the model neuron. Given that MT neuron speed tuning varies considerably, I sampled values from probability distributions that approximate neurophysiological fits to these parameters. Based on Figure 4 of [39], I sampled $\sigma_{\nu }$ from a Gaussian distribution (mean: 1.16, SD: 0.5) and $s_{0}$ from an exponential distribution (λ: 0.25/s). Consistent with Figure 8 of [39], I sampled $\nu_{pref}$ from five uniform distributions with endpoints that yielded octave spaced bins.

#### 2.3.2. Net Input

The net input of each model MT neuron is the average product of the direction and speed inputs within the RF:

(3) $$I_{MT}=\frac{1}{N_{MT,RF}}\sum_{x,y}^{N_{MT,RF}}d_{MT}\left(x,y;\theta_{pref}\right)s_{MT}\left(x,y;\nu_{pref}\right)$$

where $N_{MT,RF}$ defines the number of optic flow inputs that appear within the RF. Each MT unit (*n*) integrates the optic flow signal over time:

(4) $$\frac{dn}{dt}=-A_{MT}n+\left(B_{MT}-n\right)I_{MT}.$$

In Equation (4), $A_{MT}=0.1$ indicates the rate at which the neural response decays in the absence of input, and $B_{MT}=2.5$ indicates the excitatory upper bound of the neuron. I used Euler’s method to integrate Equation (4) with a time step of 0.1 frames (0.003 s).

#### 2.3.3. Net Activation

I applied the following sigmoid activation function to compute each MT output signal (*m*):

(5) $$m=\frac{n^{2}}{n^{2}+\gamma_{MT}^{2}}$$

where $\gamma_{MT}$ indicates the input that yields an activation value of 0.5. We set $\gamma_{MT}=0.007$, which was the approximate median *n* value on the 3D dot cloud (T) dataset.

### 2.4. Fuzzy ART Layer 1

The MT signal ($N_{MT}$x1) obtained after integrating each 10 frame optic flow sequence (Equations (4) and (5)) served as the input to the first fuzzy ART layer during training and prediction. This fuzzy ART layer consisted of 64 fuzzy ART modules arranged in a 8 × 8 spatial lattice. Each module received input from MT cells whose RF centers fall within its designated spatial sector, denoted $\vec{m}$. For example, the module on the bottom-left sector receives input from MT neurons whose RFs sample the bottom-left 64 × 64 pixel sector within each 512 × 512 optic flow frame. Due to the random placement of the MT RFs, the MT input features (*M*; length of $\vec{m}$) may differ between modules. For example, the bottom-left module may have M=113 input features if 113/5000 MT RFs sample that sector; an adjacent module may have M=97 features representing a distinct population of 97/5000 MT neurons; etc.

### 2.5. Fuzzy ART Modules

Here I summarize key stages of the fuzzy ART algorithm [32,40] implemented within each module. The input to each module was complement coded

(6) $$\vec{x}=\left[\vec{m},1-\vec{m}\right],$$

which doubles the length of each feature vector $\vec{x}$ to 2 *M*. The weights between the input and coding layers of the fuzzy ART network are initialized as a 2 $M\times C_{max}$ matrix of 1 s, where $C_{max}$ represents the maximum number of coding layer cells that may be allocated (“committed”) toward learning distinct patterns in the input. I set the value of $C_{max}$ large enough such that the network did not run out of commitable coding nodes in practice. The number of committed coding cells *C* is initially zero.

The activation $T_{j}$ of coding cell j=1,…,C, obeys the choice-by-difference (CBD) function [41,42]:

(7) $$T_{j}=\left|\right|\vec{x}\wedge {\vec{w}}_{j}{\left|\right|}_{1}+\left(1-\alpha \right)\left(M-\left|\right|{\vec{w}}_{j}{\left|\right|}_{1}\right)$$

where $\left|\right|\cdot {\left|\right|}_{1}$ indicates the $L^{1}$ norm; the ∧ operator indicates the fuzzy intersection (i.e., component-wise minimum of the two vectors) of its arguments; $\vec{w_{j}}$ is the 2M×1 weight vector associated with committed coding cell *j*; and α=0.01 balances priors on existing learned representations with their match to the current input in determining the activation $T_{j}$. The set of $T_{j}$ values is empty initially when no coding cells are committed.

#### 2.5.1. Training

The ART search cycle determines whether the weights of a previously committed coding cell will be updated based on the current input or a new coding cell will be committed during training. The search cycle checks how close the current input pattern ($\vec{x}$) is to weights of committed coding cells. Committed coding cells are checked sequentially in descending order of their activations $T_{j}$. Let *i* index the coding cell currently checked in the ART search cycle. The following function determines the degree of match between the input sample and weight vector $\vec{w_{i}}$ of coding cell *i*:

(8) $$\Gamma_{i}=\frac{\left|\right|\vec{x}\wedge \vec{w_{i}}{\left|\right|}_{1}}{\left|\right|\vec{x}{\left|\right|}_{1}}$$

If the match score meets or exceeds the “vigilance” threshold ρ (i.e., $\Gamma_{i}\geq \rho$), the weights $\vec{w_{i}}$ of the current coding cell are updated according to the following equation, and, afterwards, the next input sample is processed.

(9) $$\vec{w_{i}}=\beta \left(\vec{x}\wedge \vec{w_{i}}\right)+\left(1-\beta \right)\vec{w_{i}}$$

In Equation (9), 0≤β≤1 is a learning rate hyperparameter.

If the current coding cell does not yield a match to the input sample, the match $\Gamma_{j}$ to the next coding cell with the next highest $T_{j}$ is checked. If none of the committed coding cells yield a match or C=0 (i.e., no coding cells are yet committed), a new coding cell is committed. This means that *C* is incremented and the weight update rule (Equation (9)) is applied to $\vec{w_{C}}$, a column vector of 1 s. The next input sample is subsequently processed. I used the fast-commit slow-recode mode [32] to enhance weight stability whereby β=1 the first time a coding cell is committed and β=0.1 on subsequent weight updates.

The vigilance threshold 0≤ρ≤1 represents an important hyperparameter whose value jointly controls the granularity of learning and the number of coding cells that will be committed over the course of training. At the extremes, ρ=1 implies that separate coding cells will be committed for every input sample, while ρ=0 implies that a single coding cell will attempt to encode every sample. I constrained all modules within the same fuzzy ART layer to share a common vigilance value.

#### 2.5.2. Prediction

The output of each module is a function of the committed coding cell activations ($T_{j}$). I used the softmax function for non-output fuzzy ART layers to produce distributed, normalized activations that sum to 1. For the output layer, I selected the identity function: the raw $T_{j}$ values.

### 2.6. Non-Input Fuzzy ART Layers (MSTd Layer)

The activation of the committed coding cells across the 64 (8 × 8) modules are concatenated to form the input vector to the single module in the second fuzzy ART layer (henceforth “MSTd layer”). Because the number of committed cells is determined during training, I trained fuzzy ART layers sequentially: I trained the fuzzy ART layer 1 first, froze its weights, then trained the MSTd layer.

### 2.7. Decoding Self-Motion Estimates

I trained two decoders (one linear and one nonlinear) using gradient descent to estimate self-motion parameters from the MSTd layer activations. Both decoders were implemented as backpropagation neural networks that use the Adam optimizer with default parameters to minimize mean-squared error (MSE) loss. I standardized both the features and labels. A single-layer MLP served as the nonlinear decoder, which had 250 hidden units and the rectified-linear (ReLU) activation function. Each decoder network had two output units when estimating heading (azimuth and elevation angles) and five output units when estimating rotation (heading azimuth, heading elevation, pitch, yaw, and roll). I used early stopping (patience of 5) to halt training when the validation set loss stopped decreasing.

### 2.8. Training Protocol

I trained ARTFLOW on the training set of each dataset (Table 2) for 1 epoch by default (one-shot learning). During prediction, I froze learning and computed predicted activations on the corresponding test sets. On the neighborhood dataset, I used an 80/20 split to subdivide the 150 videos into train and test sets. The warehouse dataset served only as a test set to evaluate how well the optic flow templates learned on the neighborhood scene generalize to the novel environment.

I trained the decoders on the ARTFLOW MSTd layer activations produced to each sample in the training set after learning completed. The reported accuracy reflects decoder estimates of heading and rotation parameters from MSTd layer activations obtained to the test set samples.

### 2.9. Hierarchical Hebbian Network

I compared ARTFLOW to an identically structured hierarchical network that implements Hebbian learning rather than fuzzy ART in each module. Because the weights of a single Hebbian neuron converge to the first principal component (PC) of its input and fuzzy ART networks learn *C* optic flow templates, I implemented Sanger’s network in each module, which adapts Hebb’s Rule for learning the top *C* PCs [43]. To facilitate comparison among the networks, I configured each Sanger module to have $C_{mean}$ neurons, the mean number of committed coding cells across the corresponding fuzzy ART network layer. Unlike the fuzzy ART network, Sanger modules have the same number of neurons across the same layer.

I trained each Sanger’s network with a learning rate of 0.01. Training ceased when differences between weight matrices on successive epochs dropped below a Frobenius norm of 0.01. To prevent negativity in each neural output *x* from Sanger modules during prediction, I applied a logistic activation function:

(10) $$f\left(x\right)=\frac{1}{1+e^{-x}}$$

### 2.10. Simulation Setup and Code Availability

I implemented the model and performed simulations using MATLAB R2021b on an Apple MacBook Pro equipped with the six-core 2.9 Ghz Intel Core i9-8950HK processor and 16 GB of memory. Fuzzy ART layers were implemented such that training and prediction computations across modules ran in parallel on the CPU. The code is available on GitHub: https://github.com/owlayton/ARTFLOW (accessed on 1 October 2021).

## 3. Results

I begin by highlighting key properties of the optic flow templates learned by ARTFLOW. Subsequent sections focus on the accuracy of self-motion estimates derived from the network.

### 3.1. Learning Optic Flow Templates

**Figure 5:**
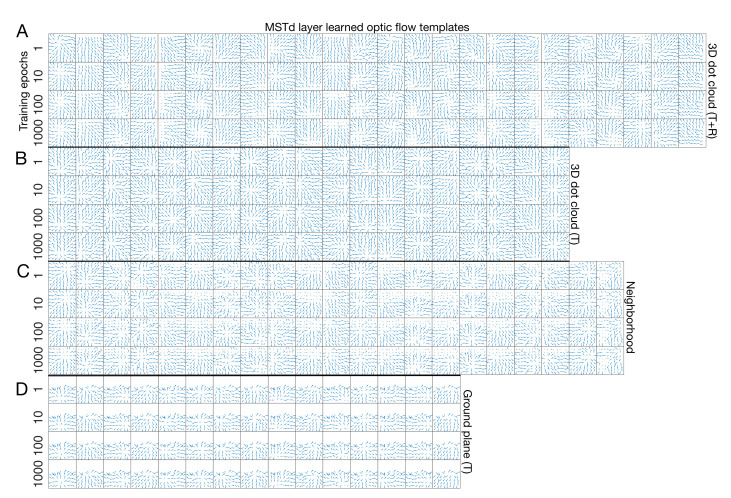
Optic flow templates learned by ARTFLOW (rows) in different visual environments (label on right-hand side). Groups of four consecutive rows correspond to each scene. Each row shows the optic flow templates after the number of training epochs indicated on the left-hand side (1 epoch is the default).

The ARTFLOW network learned diverse motion patterns that capture distinct characteristics of the optic flow encountered during simulated self-motion through each visual environment (first rows in Figure 5A–D). The optic flow templates learned from the 3D dot cloud (T + R) dataset (Figure 5A) reflect the complex spiral motion that results from combinations of translation and pitch, yaw, and roll rotation (e.g., Figure 2C). These learned patterns vary in their curvature, reflecting the differing rotation rates present across video samples. For example, some templates resemble the approximately radial optic flow that arises with smaller rotation rates, whereas others resemble the more circular patterns that may arise with larger rotation rates. When trained on optic flow from the same 3D dot cloud scene without rotation, the templates appear radial (Figure 5B) and encode a range of FoE positions. This could be viewed as an emergent basis with which to encode heading across the neural population, a hypothesis that I test quantitatively below.

Many of the learned templates from the 3D dot cloud (T) scene appear qualitatively similar to those from the neighborhood (Figure 5C) and ground plane datasets (Figure 5D), albeit in the lower portion of the visual field in the latter case. This consistency reflects the fact that the three datasets consist of full-field translational optic flow. The neighborhood templates do, however, appear less coherent due to the increased complexity of the scene and noise introduced by the optic flow estimation algorithm.

It is noteworthy that the number of optic flow templates differs across datasets without changes to the hyperparameters. This occurs because the vigilance hyperparameter (ρ) parameterizes the coarseness of learning rather than the number of cells in each ARTFLOW network layer. Consequently, the number of learned templates varies based on the statistics of each dataset and environment.

### 3.2. Stability of Learning

One key property of ART neural networks is their resilience to catastrophic forgetting, meaning that continued learning does not erode existing representations. Indeed, ARTFLOW demonstrates considerable stability in each learned optic flow template as the number of training epochs varied over several orders of magnitude (4 × 1 columns in Figure 5). The templates undergo remarkably little change from their state after a single exposure to each sample, showing the effectiveness of one-shot learning in ARTFLOW.

### 3.3. Estimating Self-Motion from Optic Flow Templates

Next, I investigated how effectively the optic flow templates learned by ARTFLOW support the accuracy of self-motion estimation. To that end, I decoded translational (heading) and rotational (pitch, roll, and yaw) self-motion parameters from MSTd layer activations after one epoch of training. I evaluated the accuracy on novel optic flow sequences not encountered during training (i.e., on distinct test sets) using linear and nonlinear (MLP-based) decoders. The performance of the ARTFLOW templates was compared with that of a principal component analysis (PCA)-based representation. This was achieved by simulating an identically structured hierarchical network that implemented Hebbian learning (Sanger’s network) in each module instead of fuzzy ART.

**Figure 6:**
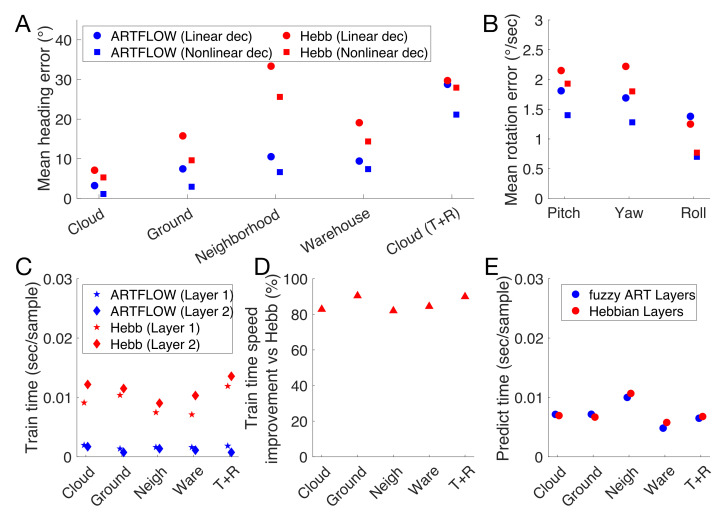
The accuracy of self-motion estimates decoded from ARTFLOW and the Hebbian networks (top row) as well as their runtimes (bottom row). (**A**) Mean absolute error (MAE) in heading estimates derived from learned optic flow templates with linear and nonlinear decoders. Values reflect error combined across azimuth and elevation. Estimates were obtained on distinct test sets not used in training. Plotted values were offset horizontally for visual clarity. (**B**) MAE in estimates of pitch, yaw, and roll rotation components on test samples from the 3D dot cloud (T + R) dataset. (**C**) Mean time required to train fuzzy ART and Hebbian layers on optic flow samples. (**D**) Same data as in (**C**) expressing the mean training time improvement in ARTFLOW compared to the Hebbian network. (**E**) Mean time required to process each optic flow sample with fuzzy ART and Hebbian layers during prediction.

Figure 6A plots the heading error decoded from the ARTFLOW and the Hebbian networks on the optic flow datasets. Regardless of the decoder, the ARTFLOW yields more accurate heading estimates than the Hebbian network, often with half the error or less. On the 3D dot cloud (T) dataset, ARTFLOW garnered a 1–3 mean absolute error (MAE), depending on the decoder, which is comparable to human performance under similar conditions [44]. The error roughly doubled on the ground dataset, which likely stems from the uncertainty caused by the lack of flow above the horizon (Figure 2B). Both networks yielded less accurate estimates on the neighborhood and warehouse datasets with optic flow derived from more realistic scenes. MAE from ARTFLOW did not exceed 10 on these two datasets, while error from the Hebbian network reached 30. Given that ARTFLOW was trained on a subset of the neighborhood optic flow samples, comparable performance on the warehouse dataset shows effective generalization of the learned templates to a novel environment.

Estimating heading in the presence of rotation (3D dot cloud (T+R) dataset) represents a much more challenging problem (Figure 1). Accordingly, both networks produced larger heading error than on the other datasets. The nonlinear decoder achieved ≈20 MAE from ARTFLOW, a substantial improvement from the ≈30 error garnered by the linear decoder. It is noteworthy that human heading estimation is also less accurate in the presence of rotation: MAE reaches 15 when estimating central headings (−4–4) with ±5/sec yaw rotation simulated in the visual display [45]. The conditions in the 3D dot cloud (T+R) dataset make heading estimation even more challenging: 1–10/sec 3D rotation and −45–45 headings. Therefore, 15 likely underestimates the error in human judgments under comparable circumstances.

Estimates of pitch and yaw rotation derived from the networks (Figure 6B) demonstrated comparable accuracy (MAE: ≈1.5–2/s). Roll was estimated with slightly higher accuracy (MAE: ≈1–1.5/s).

### 3.4. Runtime Comparison

Fuzzy ART layers in ARTFLOW required substantially less time to train than layers in the Hebbian network (Figure 6C). Figure 6D shows that this amounts to a 80–90% improvement in the training time per sample across the datasets. The time required for prediction was comparable across the networks (Figure 6E).

### 3.5. Sensitivity Analysis to Vigilance

**Figure 7:**
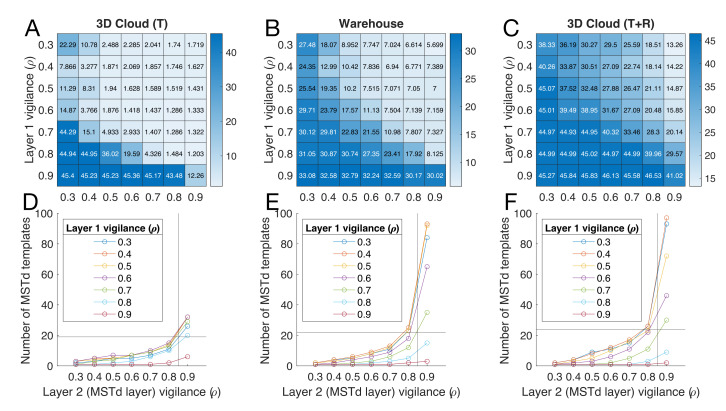
Sensitivity analysis focusing on robustness of heading estimates to the vigilance hyperparameter. (**A**–**C**) Heatmaps showing the MAE () of heading estimates obtained by the nonlinear decoder with different combinations of vigilance values in layers 1 and 2 of the ARTFLOW network on the 3D dot cloud (T), warehouse, and 3D dot cloud (T+R) datasets, respectively. (**D**–**F**) Number of optic flow templates learned by ARTFLOW on the corresponding datasets with the indicated combinations of vigilance values. Grid lines show the number of templates learned with default vigilance values.

I performed a sensitivity analysis to better understand the robustness of decoded self-motion estimates (Figure 6A,B) to changes in vigilance, a critical hyperparameter in each fuzzy ART network layer of ARTFLOW that controls the granularity of learning. Figure 7A–C show the MAE in heading estimates obtained from the nonlinear decoder with different combinations of layer 1 and 2 vigilance values. Figure 7D–F show the number of optic flow templates that emerge during training and contribute to each heading estimate. The number of templates can be viewed as the size of the basis with which self-motion is encoded. As long as the accuracy of the decoded estimate remains high, a smaller set implicates a more parsimonious encoding of self-motion and is desirable.

Figure 7A reveals that a broad range of vigilance values in both layers yields heading estimates accurate to within 1–2 on the 3D dot cloud (T) dataset. The number of templates learned from the 3D dot cloud (T) dataset remained fairly small across the range of layer 2 vigilance values tested (Figure 7D). The ≈7 MAE in heading estimates on the warehouse dataset exhibited similar tolerance to layer 1 vigilance values when coupled with moderate-to-large layer 2 vigilance values (Figure 7B). The corresponding region of the parameter space that afforded the most accurate estimates on the 3D dot cloud (T+R) was smaller (Figure 7C). ARTFLOW yielded the most accurate estimates on the warehouse and 3D dot cloud (T + R) datasets when layer 1 vigilance was low and layer 2 vigilance was high (top-right corner of Figure 7B,C). However, this comes at the cost of substantial increases in the number of optic flow templates (Figure 7E,F). The default vigilance values achieved slightly less accurate estimates with a compact set of templates, (see grid lines in Figure 7D–F).

### 3.6. Generative Model of Optic Flow

**Figure 8:**
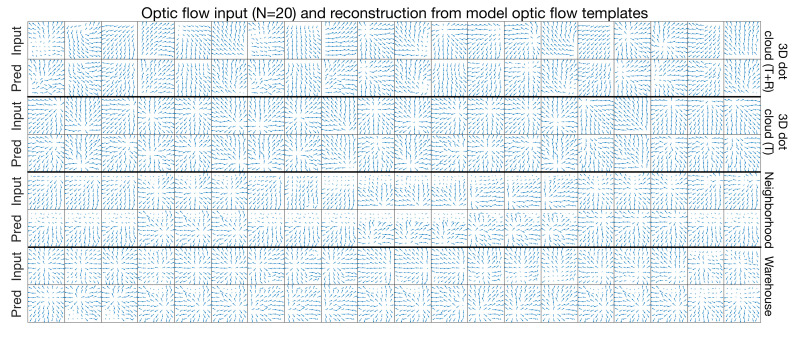
Generative capability of ARTFLOW. Twenty optic flow test samples from each dataset (“Input” rows) reconstructed by the network (“Pred” rows) based on the activation of optic flow templates.

In addition to encoding self-motion, the learned optic flow templates represent a generative model of optic flow. That is, ARTFLOW is capable of predicting the optic flow pattern that corresponds to a set of template activations. This is achieved by propagating the template activations backward layer-by-layer (see Appendix A for details). Figure 8 shows actual test optic flow samples (“Input”) and corresponding predicted patterns (“Pred”) from the activations of the ≈20 templates depicted in Figure 5. In many cases, the reconstructions emulate the singularity position and other important qualitative properties, despite not having encountered any of the test optic flow patterns during training. Deviations between the true and predicted patterns were generally largest in the neighborhood and warehouse dataset samples.

## 4. Discussion

Simulations reveal that ARTFLOW is capable of learning stable templates with only one pass through optic flow samples corresponding to simulated self-motion through a number of virtual environments (one-shot learning). This contrasts with deep learning networks that require larger amounts of data and many training epochs [46,47,48] and that suffer from the catastrophic forgetting problem [33]. While I used separate training and prediction phases in the simulations reported here, this distinction is not necessary, and ARTFLOW may continue to learn during on-going operation, unlike deep learning approaches. This offers exciting potential for adaptive, evolving sensitivity when exploring novel or changing environments.

A single hyperparameter, vigilance, controls the degree of distinction among the templates. This allows the network to self-regulate the number of templates that are needed to learn optic flow patterns with the desired granularity. Simulations revealed that the accuracy of heading estimation was robust to a wide range of vigilance values in the 3D cloud (T) and warehouse datasets (Figure 7). Sensitivity to vigilance was greater on the 3D cloud (T + R) dataset, and values that favored a larger number of templates yielded the best accuracy. Given that 3D rotation increases the dimensionality of the parameter space in which optic flow patterns may be embedded, it is indeed reasonable to expect that more neurons may be required to effectively encode self-motion. Inertial and other non-visual signals may also be used to improve self-motion estimates [16,45], perhaps without substantially increasing the number of templates in the network.

ARTFLOW not only encodes self-motion parameters more effectively than a comparable network that uses Hebbian learning (Figure 6A) but it requires far less training time (Figure 6C,D). This stems from fundamental differences in the design of the underlying algorithms. Crucially, the weight update rule in fuzzy ART modifies a single vector in the weight matrix, whereas the corresponding update in the comparable Hebbian learning algorithm (Sanger’s network) requires three separate matrix multiplications to combine the weight matrix with with lower triangular portion of the squared activation matrix [43]. While fuzzy ART relies on an iterative search process for selecting the best matching template during learning, it amounts to checking scalar values that represent the degree of match to each template at most once, in the worst case. By contrast, training with the Hebbian learning algorithm tends to involve iterative optimization over multiple training epochs.

### Comparison to Other Models

Several neural networks have been developed that adaptively learn the self-motion templates using Hebbian or Instar biological learning laws [49,50,51]. However, their focus is on characterizing neurophysiological properties of MSTd rather than estimating self-motion from optic flow extracted from video. As I demonstrated here, such networks are unlikely to perform as well as ARTFLOW. Beyeler and colleagues have introduced a model of MSTd that uses non-negative matrix factorization to learn optic flow templates [38]. While the model captures an extensive collection of neurophysiological properties of MSTd, it does not specify the neural network mechanisms for the underlying computations and requires iterative optimization (i.e., multiple training epochs). The model yields 5.75 error when estimating heading over the $\left(-45,45\right)$ range considered here from a dot-defined environment that consists of intersecting ground and frontoparallel wall planes. This environment was not simulated here, but it bears the most similarity to the 3D cloud and ground plane datasets on which ARTFLOW garnered 3.24 and 7.46 heading error, respectively. It should be noted that ARTFLOW achieves this level of accuracy with 500 optic flow training samples and ≈20 learned templates, compared to the 10,000 training samples and 144 templates used by the Beyeler model. If the two models achieve comparable accuracy on the ground + wall dataset, ARTFLOW would do so with substantially less training and a more compact representation.

ARTFLOW complements work others have done to develop fuzzy ART into a multi-layer network. ARTree [52,53] is a hierarchical network that differs most substantially from ARTFLOW in its coarse-to-fine structure: a single fuzzy ART network processes each data sample in the first layer, and the number of modules increases with each successive layer. Distributed dual-vigilance fuzzy ART (DDVFA) contains sets of modules that process information globally and locally at the same hierarchical level [40]. Other approaches [54,55] successively pass the entire output of a fuzzy ART network as features to another fuzzy ART network. This would approximate chains of fuzzy ART layers in ARTFLOW configured as single 1 × 1 modules. Like MLPs, this implicates global pattern processing without consideration of the local structure of data. It is unclear how suitable these alternative approaches are for processing optic flow or large volumes of other sensory data.

## 5. Conclusions

Algorithms inspired by the design of the brain have the potential to bridge the gap between machines and animals when it comes to agile, resilient, adaptive navigation. Toward that end, I have presented ARTFLOW, a fast, unsupervised biologically inspired neural network that learns optic flow patterns that support accurate self-motion estimation in diverse environments. The network demonstrates effective encoding of self-motion parameters with one-shot learning and representations that remain stable over continued training.

## Figures and Tables

**Table 1:** Parameters specifying self-motion through 3D dot cloud and ground plane environments.

Parameter	Value
Video spatial resolution	512 × 512 pixels
Translational speed	3 m/s
Camera focal length	1.74 cm
Field of view	90
Eye height	1.61 m
Number of dots in scene	2000
Observer-relative depth range of dots	1–50 m

**Table 2:** Specification of datasets with dot-defined environments. *T* indicates optic flow that contains only translation, and T+R indicates optic flow with both translational and rotational components. Training set is used to learn optic flow templates, while test set is used to evaluate performance on novel patterns. Uniform indicates sampling from the uniform random distribution with the specified endpoints. Azimuth and elevation angles of 0 correspond to straight-ahead heading.

Dataset	Description	Size (Num Videos)	Independent Variables
3D dot cloud (*T*)	Translational self-motion through 3D dot cloud along random heading directions	Train: 500 Test: 250	Heading azimuth and elevation: Uniform(−45°,45°)
Ground plane (*T*)	Translational self-motion over a ground plane with random heading directions	Train: 500 Test: 250	Heading azimuth and elevation: Uniform(−45°,45°)
3D dot cloud (T+R)	Self-motion through 3D dot cloud along random 2D heading directions with varying amounts of pitch, yaw, roll rotation	Train: 1000 Test: 500	Heading azimuth and elevation: Uniform(−45°,45°) Rotation speed: Uniform(1,10)°/s

**Table 3:** ARTFLOW hyperparameters and default values used in simulations. Most MT hyperparameters are set according to physiologically informed values.

Parameter	Layer Type	Description	Value
$N_{MT}$	MT	Number of MT neurons	5000
	MT	RF radius	15 pixels (2.5°)
$\theta_{pref}$	MT	Preferred direction of neuron	Uniform (0°, 360°)
$\sigma_{\theta }$	MT	Bandwidth of direction tuning	3°
$\sigma_{\nu }$	MT	speed tuning bandwidth	$N\left(\mu_{s}=1.16{}^{\circ},\sigma_{s}=0.5{}^{\circ}\right)$
$s_{0}$	MT	Non-negative offset parameter to prevent singularity in speed logarithm	Exp $\left(0.25{}^{\circ}/s\right)$
$\nu_{pref}$	MT	Preferred speed of neuron	Uniform $\left(0.5,2.0\right){}^{\circ}/s$, Uniform $\left(2.0,4.3\right){}^{\circ}/s$, Uniform $\left(4.3,7.6\right){}^{\circ}/s$, Uniform $\left(7.6,12.7\right){}^{\circ}/s$, Uniform $\left(12.7,32.0\right){}^{\circ}/s$
$A_{MT}$	MT	Passive decay rate	0.1
$B_{MT}$	MT	Excitatory upper bound	2.5
	MT	Temporal integration time step	0.1 frames (0.003 s)
$\gamma_{MT}$	MT	Input value that yields 0.5 in sigmoid activation function	0.007 (median empirical input)
	Fuzzy ART	Fuzzy ART module arrangement	Layer 1: 8 × 8, layer 2: 1 × 1
α	Fuzzy ART	Activation prior on existing representations	0.01
ρ	Fuzzy ART	Vigilance	Layer 1: 0.65, layer 2: 0.85
β	Fuzzy ART	Learning rate	1 (when committing new cell)0.1 (when updating weights)
	Fuzzy ART	Number of training epochs	Layer 1: 1, layer 2: 1

## Abbreviations

The following abbreviations are used in this manuscript:

SD	standard deviation
T	translation
R	rotation
MSE	mean squared error
MAE	mean absolute error
MSTd	dorsal medial superior temporal area
MT	medial temporal area
PCA	principal component analysis
RF	receptive field

## Appendix A. Generative Model of Optic Flow

To predict the optic flow that corresponds to activations in the top “MSTd” layer of ARTFLOW, learned optic flow templates send signals backward through the network until they reach the MT preprocessing layer. At this point the signals may be decoded within each module according to known motion tuning properties of MT neurons. This process estimates a single optic flow vector that captures the average (centroid) direction within the RF of each module in the first fuzzy ART layer. That is, optic flow is estimated at the spatial resolution of the modules in the first layer of the network (e.g., 8 × 8 for 8 × 8 modules).

Let $M_{upper}$ denote the number of committed cells in a single module of an “upper fuzzy ART layer” of the network and $M_{lower}$ denote the total number of committed cells across the connected modules in the adjacent fuzzy ART layer directly beneath. For example, if the upper layer represents the top MSTd layer with a single module that contains 20 learned optic flow templates then $M_{upper}=20$. If the upper layer module is connected with four modules in the layer below, each containing 10 committed cells, then $M_{lower}=40$. The activation ${\mathrm{act}}_{j}^{lower}$ of each cell $j=1,\ldots ,M_{lower}$ in the lower layer based on the descending top-down signals from the upper layer module is

(A1) $${\mathrm{act}}_{j}^{lower}=\sum_{i=1}^{M_{upper}}w_{ji}{\mathrm{act}}_{i}^{upper}$$

where *w* represents the weights learned between the upper fuzzy ART module and committed cells in the connected lower modules (shape: $M_{lower}$×$M_{upper}$). This computation is repeated for all modules in the lower layer and for any fuzzy ART layers beneath the lower layer (i.e., lower layer subsequently becomes the upper layer).

At the MT preprocessing layer, the direction of optic flow within the RF of each module is estimated according to population vector (centroid) decoding [56]. Let $N_{s}$ represent the number of MT neurons within the rectangular spatial sector *s*, the RF of module *s* (pink squares in Figure 2). The horizontal $u_{s}$ and vertical $v_{s}$ estimated optic flow vector components in the sector are given by

(A2) $$\left(u_{s},v_{s}\right)=\frac{1}{\sum_{i=1}^{N_{s}}act_{i}}\sum_{i=1}^{N_{s}}\left(act_{i}cos\left(\theta_{pref,i}\right),act_{i}sin\left(\theta_{pref,i}\right)\right)$$

where $act_{i}$ represents the activation of MT neuron *i* in the sector *s* due to the top-down descending signals, and $\theta_{pref,i}$ is the preferred direction of the MT neuron.
